# Exploring the Effects of Meditation Techniques Used by Mindfulness-Based Programs on the Cognitive, Social-Emotional, and Academic Skills of Children: A Systematic Review

**DOI:** 10.3389/fpsyg.2021.660650

**Published:** 2021-11-12

**Authors:** Marisa G. Filipe, Sofia Magalhães, Andreia S. Veloso, Ana Filipa Costa, Lúcia Ribeiro, Patrícia Araújo, São Luís Castro, Teresa Limpo

**Affiliations:** ^1^Center of Linguistics, School of Arts and Humanities, University of Lisbon, Lisbon, Portugal; ^2^Center for Psychology at University of Porto, Faculty of Psychology and Education Sciences, University of Porto, Porto, Portugal; ^3^TRIE-Transdisciplinary Research Center for Innovation & Entrepreneur Ecosystems, Manuel Teixeira Gomes Higher Education Institute (ISMAT), Portimão, Portugal

**Keywords:** meditation, mindfulness, mindfulness techniques, children, systematic review

## Abstract

There is evidence for the positive impact of mindfulness in children. However, little is known about the techniques through which mindfulness practice results in differential outcomes. Therefore, this study intended to systematically review the available evidence about the efficacy of meditation techniques used by mindfulness-based programs on cognitive, socio-emotional, and academic skills of children from 6 to 12 years of age. The review was registered on the PROSPERO database, and the literature search was conducted according to PICO criteria and PRISMA guidelines. The EBSCO databases were searched, and 29 studies were eligible: nine randomized controlled trials and 20 quasi-experimental studies. All the included randomized controlled trials were rated as having a high risk of bias. Overall, the evidence for mindfulness techniques improving cognitive and socio-emotional skills was reasonably strong. Specifically, for cognitive skills, results showed that all the interventions used “body-centered meditations” and “mindful observations.” Regarding socio-emotional skills, although all the studies applied “body-centered meditations” and “mindful observations,” “affect-centered meditations” were also frequent. For academic skills, just one quasi-experimental trial found improvements, thus making it difficult to draw conclusions. Further research is crucial to evaluate the unique effects of different meditation techniques on the cognitive, social-emotional, and academic skills of children.

**Systematic Review Registration:** Identifier: RD42019126767.

## Introduction

“Mindfulness” is a term frequently used to describe a mental faculty related to attention, awareness, retention/memory, and/or discernment (Davidson and Kaszniak, 2015). A popular definition of mindfulness entails a deliberate conscious awareness of the present moment without judgment (Kabat-Zinn, 2003). Another common meaning of mindfulness is related to a specific form of meditative practice (Goleman, 1988; Goleman and Davidson, 2018). This type of meditation fosters the ability to bring a non-judging awareness to a specific thing and strengthens our ability to notice our mind wandering (Goleman and Davidson, 2018). Importantly, mindfulness is a psychological process that can be developed through practice (Kabat-Zinn, 2003; Bishop et al., 2004), and several studies have shown that this specific training leads to improvements in psychological wellbeing and mental health (e.g., Bowen et al., 2006; Chiesa, 2009; Chiesa and Serretti, 2011; for a review see Keng et al., 2011). These positive effects have also been highlighted among children (for a review and meta-analyses see Klingbeil et al., 2017 and Maynard et al., 2017). Still, little is known about the techniques through which mindfulness practice results in those benefits, particularly for children. Thus, in this study, we performed a systematic review of the current literature about the efficacy of meditation techniques used by mindfulness-based programs on the cognitive, social-emotional, and academic skills of children.

Mindfulness-based interventions employ several training techniques (Kabat-Zinn, 2003), which involve a huge number of diverse practices. To do justice to this diversity, several attempts were made to classify these practices, and different taxonomies included a diversity of techniques from various backgrounds and contexts (Nash and Newberg, 2013; Schmidt, 2014; Lutz et al., 2015). For instance, Singer et al. (2016) developed a classification of practices based on Buddhist traditions, contemplative sciences, and neuroscientific research. The authors distinguished three broad classes of mental skills: (1) present-moment attention and body awareness, including breathing meditation and body scan as core exercises; (2) socio-affective abilities such as gratitude, compassion, prosocial motivation, and accepting difficult emotions through loving-kindness meditation and dyadic exercises as core practices; and (3) socio-cognitive capacities such as metacognition and perspective taking that incorporates core practices like observing thoughts, meditation, and dyadic perspective-taking exercises. Furthermore, Hildebrandt et al. (2017) explored the differential effects of these classes of mental abilities and found that present-moment attention practices increased attentional facets, but only socio-affective and socio-cognitive training led to broad changes in ethical-motivational skills such as nonjudgmental attitude, compassion, and self-compassion.

In 2019, Matko and Sedlmeier developed a new classification system for meditation techniques to make it accessible and understandable to practitioners/researchers with different backgrounds. Through a survey with 100 experienced meditators, the authors found seven main clusters of techniques: (1) body-centered meditation (i.e., concentrating on energy center or channeling, body scan, breath abdomen, observing the body, and breath nose); (2) mindful observation (i.e., observation of thoughts or emotions, long meditation, and sitting in silence); (3) contemplation (i.e., contemplating on a question, contradiction, or paradox); (4) mantra meditation (meditation with sound, singing sutras or mantras, and repeating syllables); (5) visual concentration (i.e., visualizations and concentrating on an object); (6) affect-centered meditation (i.e., cultivating compassion and opening up to blessings); and (7) meditation with movement (i.e., meditation with movement, manipulating the breath, walking, and observing senses).

Several reviews have examined the efficacy of mindfulness practice on adults, and there is evidence for the positive impacts of mindfulness training (Baer, 2003; Grossman et al., 2004; Mackenzie et al., 2005; Smith et al., 2005; Ott et al., 2006; Matchim and Armer, 2007; Toneatto and Nguyen, 2007; Winbush et al., 2007; Praissman, 2008; Teixeira, 2008; Carmody and Baer, 2009; Ledesma and Kumano, 2009). Some of these reviews applied meta-analytic methods to quantify the efficacy of this intervention (Baer, 2003; Grossman et al., 2004; Ledesma and Kumano, 2009), and robust evidence for the positive impact of mindfulness practice was found. Research with children is not yet as extensive as with adults, but it is growing rapidly. For example, two recent meta-analyses indicated the increased interest in the utility of mindfulness training in young people. Specifically, Klingbeil et al. (2017) reported data from participants between 4 and 18 years of age and analyzed two broad categories of outcome measures: (i) skills of mindfulness, attention, and meta-cognition/cognitive flexibility and (ii) academic performance and emotional/behavioral regulation. As a result, the authors found significant improvements across outcomes in all categories. Maynard et al. (2017) analyzed data from studies implementing mindfulness training in schools to participants aged between 4 and 20 years and also found that this intervention had a small-to-medium effect on cognitive and socio-emotional skills.

Research has repeatedly shown that mindfulness training improves the performance of children on tasks that assess cognitive functioning such as attention or executive functions (e.g., Semple et al., 2010; Leonard et al., 2013; Britton et al., 2014; Schonert-Reichl et al., 2015; Felver et al., 2017; Lawler et al., 2019). For example, 12 sessions of mindfulness training improved the performance of preschoolers on an attention task, while no changes were observed in the passive control group (Quan et al., 2019). For executive functions, after 8 weeks of mindfulness training, parents reported improvements in children in inhibition, shift, emotional control, initiative, working memory, planning, organization of materials, and monitoring skills (7–9 years old) (Flook et al., 2010).

The impact of mindfulness training has also been studied on emotional mental health (Bohlmeijer et al., 2010; Fjorback et al., 2011; Gotink et al., 2015; Guendelman et al., 2017). For example, after 12 weeks of mindfulness, yoga movements, and breathing training, fourth- and fifth-grade students (i.e., 9- and 10-year-old children) reduced involuntary responses to stress (such as rumination and intrusive thoughts) when compared with a waitlist control group (Mendelson et al., 2010). Napoli et al. (2005) examined the effects of a 24-week mindfulness intervention on attention and anxiety levels in first- and third-grade students, and results showed that mindfulness training reduced attentional problems and anxiety in children. Furthermore, mindfulness interventions have been found to improve social-emotional skills. For instance, teachers reported significant increases in optimism and improvements on classroom social behaviors of students (9–13 years of age) who participated in a mindfulness education program (Schonert-Reichl and Lawlor, 2010).

As mindfulness has shown positive effects on many aspects of wellbeing, studying the impact of mindfulness in schools has been worth it (Huppert and Johnson, 2010). For instance, studies have found a positive relationship between mindfulness and academic performance (McCloskey, 2015; Lin and Mai, 2018), probably because it reduces stress and anxiety (McCloskey, 2015), increases attention and memory (Lin and Mai, 2018), and/or enhances specific skills such as openness, attention, or inquiry (Docksai, 2013). As an example, performance on a reading comprehension test was significantly improved after participation in an intensive 2-week mindfulness training (Mrazek et al., 2013).

Even though research has accumulated evidence suggesting that mindfulness training improves cognitive, socio-emotional, and academic skills, studies are needed to clarify which intervention techniques produce change (Shapiro and Carlson, 2009). Yet, a systematic review focusing on the meditation techniques used by mindfulness-based programs that result in those outcomes has not been conducted. Thus, our primary aim was to identify different techniques to improve cognitive, social-emotional, and academic outcomes. Specifically, we explored a multiplicity of meditation-based techniques. As research with children is growing rapidly, we focused our attention on school-age children. We also selected other study inclusion criteria to maintain the focus on interventions delivered with high quality, i.e., the studies should include an active or an inactive/passive control condition to provide a comparable condition and quantitative measures should be reported as outcomes. We expected to analyze the frequency and impact of different techniques in order to understand which practices are frequently used for promoting different types of skills. Our conclusions are likely to reveal how future research on the effectiveness of mindfulness interventions may be improved.

## Method

### Search Strategy

A systematic literature search was carried out using MEDLINE and EBSCO (PsycINFO, CINAHL, ERIC) databases from the year 2009 to March 2019. An update was conducted from the year 2019 to March 2021. The review was conducted according to PRISMA guidelines (Moher et al., 2009) and was registered on the PROSPERO database for systematic reviews (registration 2019: CRD42019126767). The following keywords were used to conduct the search: Child^*^ OR Children^*^ OR “Primary School” OR “Elementary School” OR “Primary Education” OR “Elementary Education” AND (Mindfulness^*^ OR Mindful^*^) AND (Intervention^*^ OR Training^*^ OR Program^*^ OR Exercise^*^ OR Techniques^*^) NOT (“Clinical Population” OR “Clinical Patients” OR Patients^*^ OR Clinical^*^ OR Inpatient^*^ OR Outpatient^*^ OR Disorder^*^ OR Disabilities^*^). Filters for source types (academic Journals), age (6–12 years), and language (English) were applied. An additional search through other sources was conducted.

### Inclusion and Exclusion Criteria

Using the Population, Interventions, Comparison, Outcomes (PICO) framework, inclusion and exclusion criteria were based on the following research question: In typical school-age children, what meditation techniques used by mindfulness-based programs, compared with other types of intervention and/or placebo conditions, are more effective in developing cognitive, social-emotional, and academic outcomes? (Table 1).

**Table 1:** Population/participant, intervention/indicator, comparator/control, outcome (PICO) framework.

**PICO framework**	
Population	Typically developing children from 6 to 12 years of age
Intervention	Meditation techniques used by mindfulness-based programs
Comparison	Other types of intervention and/or a placebo condition
Outcome	Cognitive, social-emotional, and academic outcomes

In order to be included, studies had to: (i) include typically developing children aged 6–12 years; (ii) clearly describe the mindfulness techniques employed; (iii) include an active or an inactive/passive control condition to provide a comparable condition to test the effects of techniques; (iv) include measures of cognitive, socio-emotional, or academic skills as outcomes; (v) provide quantitative measures; and (vi) be published in English. Reviews, meta-analyses, editorials, opinion papers, and dissertations were excluded.

### Risk of Bias (Quality) Assessment

To assess the risk of bias in randomized trials, we used the Cochrane Collaboration's tool, namely, the RoB 2.0 (Sterne et al., 2019). This tool assesses five domains of bias: (1) bias due to randomization, (2) bias due to deviations from intended intervention, (3) bias due to missing data, (4) bias due to outcome measurement, and (5) bias due to selection of the reported result. The risk of bias was assessed by two authors (AV and SM) independently from each other (selecting “low risk,” “high risk,” or “no information” of bias). The unclear risk was selected when details were not reported or unknown. Discrepancies were resolved through discussion. Given that we anticipated that most of the studies would be at high risk of bias, we did not restrict analyses based on this parameter.

### Data Extraction

We developed the data extraction from all eligible articles based on the Preferred Reporting Items for Systematic review and Meta-Analysis Protocols (PRISMA-P; Moher et al., 2009) flow diagram, following four stages: (1) identification, (2) screening, (3) eligibility, and (4) inclusion. Once the references had been obtained, we used the Rayyan software (Ouzzani et al., 2016) to compile the articles. Studies were initially identified by title and abstract, according to the inclusion criteria established. This full search was evaluated for inclusion by two authors (SM and AF) independently from each other, and discrepancies were resolved by discussion. A study was included when both reviewers independently assess it as satisfying the inclusion criteria. A third author (MF) mediated in the event of disagreement following discussion. Extracted information included: studies characteristics (*viz*. general study characteristics, effects of interventions, and mindfulness techniques). The types of techniques used within each intervention were coded according to the seven main clusters suggested by Matko and Sedlmeier (2019).

## Results

### Trial Flow

**Figure 1:**
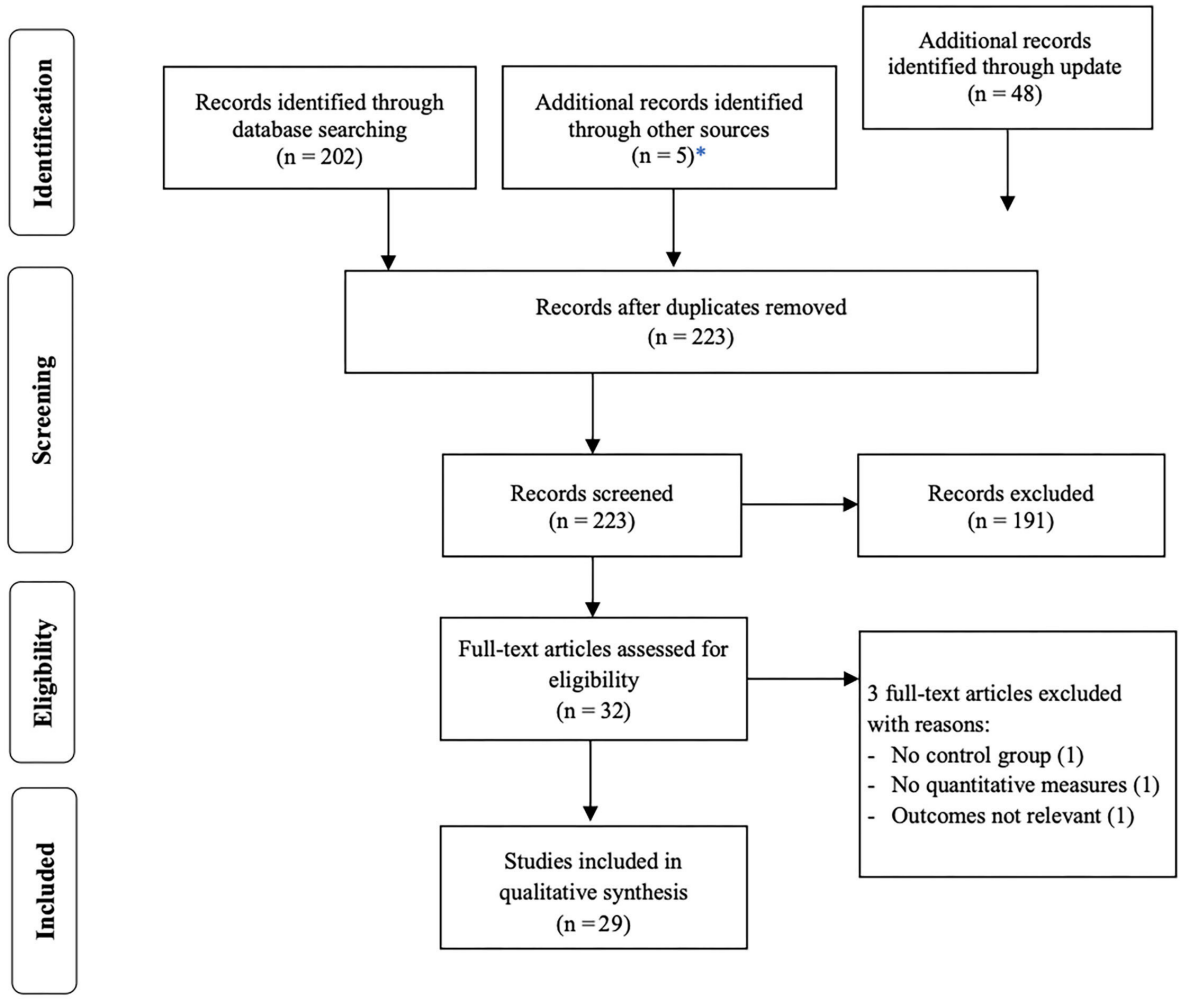
Preferred Reporting Items in Systematic Reviews and Meta-Analyses (PRISMA) flow diagram of selection of studies. ^*^Other sources for identification of further articles included Google Scholar and “snowballing” techniques such as searching non-indexed journals and references of all full-text articles.

A total of 250 articles were identified from the databases using the search strategy previously described. Five articles were added through other sources. Thirty-two duplicates were removed, and 223 articles were screened by title and abstract. Of these, 191 reports were excluded since they did not meet the inclusion criteria. There was almost perfect agreement between the two judges (Cohen's κ = 0.97). Thus, 32 papers were included, and their full-text analyzed, of which 29 met inclusion criteria. This trial flow is presented in a PRISMA flow diagram in Figure 1.

### General Study Characteristics

#### Settings

Twelve of the 29 studies included in this review were conducted in the United States. The other studies were conducted in Canada (*n* = 2), Israel (*n* = 2), United Kingdom (*n* = 2), Spain (*n* = 2), Australia (*n* = 1), Brazil (*n* = 1), Germany (*n* = 1), Korea (*n* = 1), Italy (*n* = 1), Netherlands (*n* = 1), New Zealand (*n* = 1), Philippines (*n* = 1), and Portugal (*n* = 1) (Table 1). Four studies were published between 2010 and 2012, seven studies were published between 2014 and 2015, while 15 studies were published between 2016 and 2018, and three studies were published between 2019 and 2021 (Table 2).

**Table 2:** Characteristics of the included studies.

**Authors**	**Country**	**Method**	**Age range**	**Type of comparison groups**	**Comparison groups conditions**	**Intervention group(*n*)**	**Comparison groups (*n*)**
Alampay et al. (2019)	Philippines	RCT	9–16 years	Active	Handicrafts condition	87	99
Bakosh et al. (2016)	United States	QED	3rd grade	Passive	Business as usual	93	98
Bakosh et al. (2018)	United States	RCT	1st−4th grades	Passive	Waitlist	167	170
Bauer et al. (2020)	United States	RCT	Mean = 11.76 years (SD = 0.40)	Active	Coding training	15	16
Britton et al. (2014)	United States	RCT	Mean = 11.79 years, (SD = 0.41)	Active	African history course with a matched experiential activity	55	46
Crescentini et al. (2016)	Italy	QED	7–8 years	Active	Emotion awareness not involving meditation exercises	16	15
Bergen-Cico et al. (2015)	United States	QED	6th grade	Active	Exposure to information about mindful awareness, but do not practice mindful yoga and meditation	72	72
Butzer et al. (2017)	United States	RCT	7th−12 th grade	Active	Physical education as usual	117	94
de Carvalho et al. (2017)	Portugal	QED	3rd−4th grade	Passive	Waitlist	223	231
Devcich et al. (2017)	New Zealand	QED	9–11 years	Active	Emotional literacy program	54	52
Enoch and Dixon (2017)	United States	QED	6–12 years	Passive	Business as usual	20	20
Flook et al. (2010)	United States	RCT	7–9 years	Active	Silent reading period	32	32
Gould et al. (2012)	United States	QED	4th−5 th grade	Passive	Waitlist	51	46
Janz et al. (2019)	Australia	QED	Mean = 78.03 months (SD = 10.71)	Passive	Waitlist	55	36
Parker et al. (2014)	United States	RCT	9–11 years	Passive	Waitlist	71	40
Ricarte et al. (2015)	Spain	QED	6–13 years	Passive	Waitlist	45	45
Rodríguez-Ledo et al. (2018)	Spain	QED	11–14 years	Passive	Business as usual	108	48
Schonert-Reichl and Lawlor (2010)	Canada	QED	9–13 years	Passive	Waitlist	139	107
Schonert-Reichl et al. (2015)	Canada	RCT	9–11 years	Active	Social responsibility program	48	51
Tarrasch (2018)	Israel	QED	9–10 years	Passive	Business as usual	58	43
Tarrasch et al. (2017)	Israel	QED	4th−5th grade	Passive	Waitlist	138	78
Thomas and Atkinson (2016)	United Kingdom	RCT	8–9 years	Passive	Waitlist	16	14
van de Weijer-Bergsma et al. (2014)	Netherlands	QED	8–12 years	Passive	Waitlist	95	104
Viafora et al. (2015)	United States	QED	11–13 years	Passive	Waitlist	Group 1: 28Group 2: 15	20
Vickery and Dorjee (2016)	United Kingdom	QED	7–9 years	Passive	Waitlist	33	38
Waldemar et al. (2016)	Brazil	QED	10–14 years	Passive	Waitlist	64	68
White (2012)	United States	QED	8–11 years	Passive	Waitlist	70	85
Wimmer et al. (2016)	Germany	QED	5 th grade	1 Active1 Passive	Concentration training; Business as usual	16	Group 1: 8 Group 2: 10
Yook et al. (2017)	Korea	QED	2nd−4 th grade	Passive	Business as usual	23	23

*RCT, randomized controlled trial; QED, quasi-experimental design*.

#### Participants

Age varied across studies. Four studies included 6-year-olds, nine studies recruited 7-year-olds, 13 studies recruited 8-year-olds, 19 studies recruited children with 9 years old, 16 studies included children with 10 years old, and 15 studies included children with 11 years old (Table 2). Sample sizes for intervention groups ranged from 15 to 223 and for control groups from 8 to 231.

#### Research Design

Nine randomized controlled trials (RCT) and 20 quasi-experimental design (QED; studies using a comparison group design, but participants were not randomly assigned to conditions) were included in the review (Table 2). Regarding control groups, only one study compared the performance of the mindful group with an active and a passive control group (i.e., concentration training and business as usual, respectively). Nineteen studies included only passive control groups (i.e., waitlist and business as usual) and nine included only active control groups (i.e., handcrafts condition, coding training, history course, emotional literacy program, silent reading period, social responsibility program, physical education, and exposure to mindful awareness; Table 2).

#### Risk of Bias

**Figure 2:**
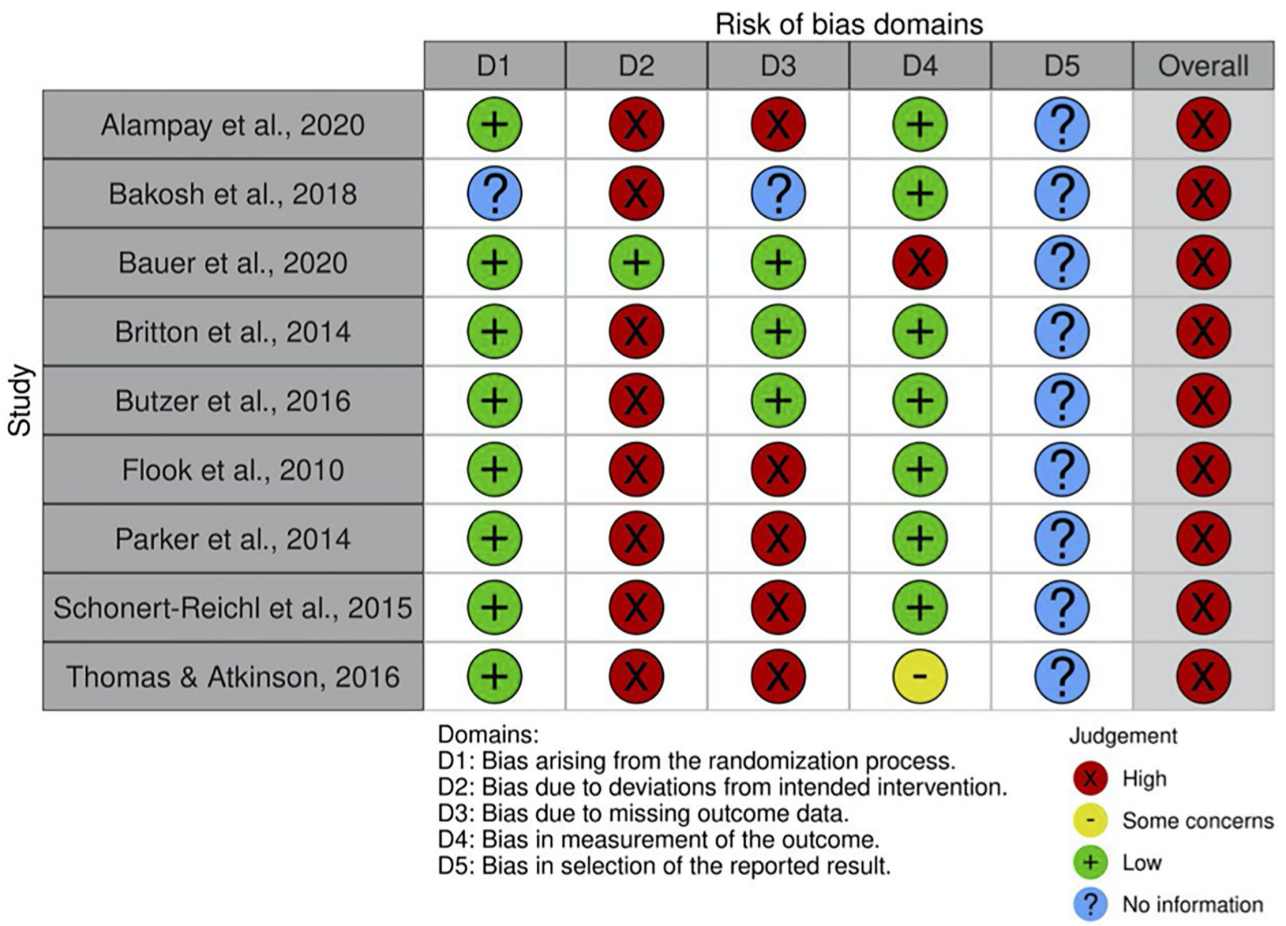
Risk of bias graph across randomized controlled trials.

**Figure 3:**
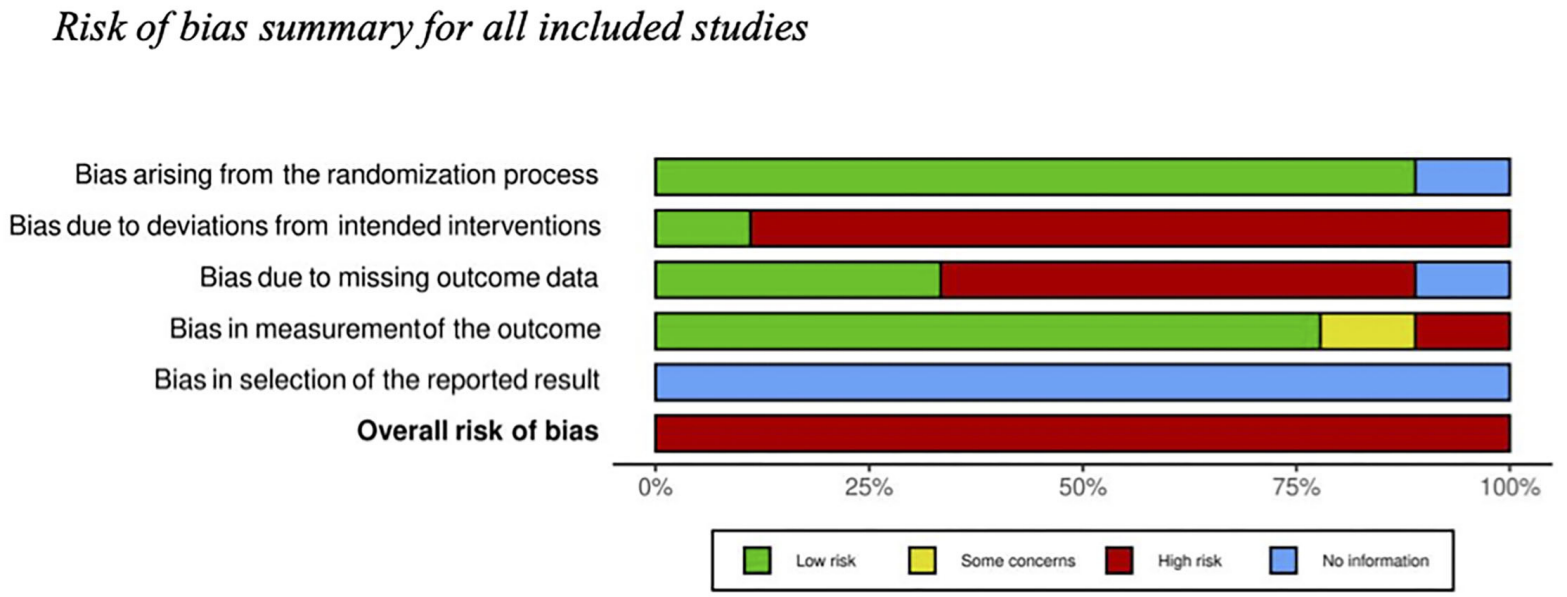
Risk of bias summary across randomized controlled trials.

Several authors fail to report design characteristics (i.e., allocation concealment, blinding, and incomplete outcome data) to conduct an accurate assessment of the risk of bias. There was almost perfect agreement between the two judges that conduct the assessment (Cohen's κ = 0.89). Overall, there was a high-risk of bias across the 9 RCTs included in the review, with variation in high-risk areas across studies see Figure 2 for a table reporting each domain of risk for each study and Figure 3 for a summary of risk across studies.

#### Intervention Characteristics

Duration, intensity, and dosage of mindful interventions varied across the 29 included studies. For studies reporting adequate information, a wide range of daily doses of formal mindfulness practice were reported, from 4 to 90 min per session. Interventions ranged from 2 weeks to 9 months. Studies also varied in terms of how frequently children met to receive the intervention, from one time per week to daily interventions. The most frequent option was sessions of 45 min once a week for 8 weeks (Table 3).

**Table 3:** Intervention characteristics, meditation techniques, and main findings of the included studies.

**Study**	**Intervention**	**Duration/Intensity**	**Targets of intervention**	**Main findings**
Alampay et al. (2019)	Mindfulness-based cognitive therapy (MBCT) adapted to the Kamalayan curriculum.	10 sessions of 75 min for the younger students or 90 min for the older students (10 weeks)	Depressive and anxiety symptoms and emotion regulation	Results indicated that participation in the mindful group did not affect depression, anxiety, or emotion regulation.
Bakosh et al. (2016)	Mindful-based social and emotional learning (MBSEL)	40 sessions of 10 min-per-day (8 weeks)	Students' grades (reading, science, math, writing, spelling, and social studies), classroom behavior, day-to-day teaching operations	The mindful training significantly predicted a difference in science and reading grades. This experimental group also showed improvements in classroom behavior, compared with the control group.
Bakosh et al. (2018)	Mindfulness-based stress reduction (MBSR)–adapted program	10-min-per-day audio-guided mindfulness program (10 weeks)	Academic achievement	Results showed that improvements in Math scores, Social Studies scores, and Grade Point Averages were generally higher for students in the intervention group. However, the results varied considerably in effects and there is a lack of consistent statistically significant results.
Bauer et al. (2020)	Calmer Choice	4 sessions of 45 min each per week (8 weeks)	Sustained attention and associated resting-state functional brain connectivity (i.e., anticorrelation between the default mode network [DMN] and right dorsolateral prefrontal cortex [DLPFC])	Participants in the mindful group preserved fewer lapses of attention and DMN–DLPFC anticorrelation (associated with better performance on a sustained-attention task) compared to children in the control group.
Britton et al. (2014)	Meditation condition formulated according to Roth's Integrative Contemplative Pedagogy	3 to 12 min each day (6 weeks)	Behavioral and emotional problems	Both groups decreased significantly on clinical syndrome subscales and affect but did not differ in the extent of their improvements. The mindful group was significantly less likely to develop suicidal ideation or thoughts of self-harm than controls.
Crescentini et al. (2016)	Mindfulness-oriented meditation based on the mindfulness-based stress reduction protocol	3 sessions per week (10 min - 1 h each) (8 weeks)	Cognitive, emotional, social, and behavioral processes	The mindful group showed positive effects in reducing attention problems. Both groups reduced their internalizing problems such as anxiety.
Bergen-Cico et al. (2015)	Yoga mindful intervention inspired by YogaKids	3 sessions of 4 min each per week (100 school days)	Self-regulation	The mindfulness group showed improvements in long-term and global self-regulation, compared with the control group.
Butzer et al. (2017)	Kripalu Yoga in the Schools (KYIS) curriculum	1 or 2 sessions of 45 min per week (32 sessions, 6 months)	Substance use willingness, actual substance abuse, emotional self-regulation, perceived stress, mood, and impulsivity	The mindfulness group showed improvements in their willingness to smoke cigarettes as well as improvements in emotional self-control in females), compared with the control group.
de Carvalho et al. (2017)	MindUP program	15 sessions of 45-60 min each + 3 min of meditation every day, 3 times a day (15 weeks)	Children's emotional regulations skills, self-compassion, and affect teachers' mindfulness, self-compassion, emotion regulation skills, and burnout	In the group of children: The mindful group demonstrated higher improvements, than the control group, in social and emotional skills, namely in positive emotions, common humanity (a dimension of self-compassion), and a significant reduction in suppressing their emotions. In the group of children teachers: The mindful group showed superior improvements than the control group, in self-kindness, personal accomplishment, and observation abilities.
Devcich et al. (2017)	Pause, Breath, Smile	1 session of 60 min per week (8 weeks)	Self-reported wellbeing (including components of subjective wellbeing and psychological wellbeing) and mindfulness	The mindfulness group showed significant increases in self-reported wellbeing, compared with the control group. Mindfulness scores were significantly increased only for the mindfulness group.
Enoch and Dixon (2017)	Acceptance and commitment therapy curriculum	6 sessions of 20 min each (2 weeks)	Attention processes	The mindful group showed that increases in attention outcomes, compared with the control group.
Flook et al. (2010)	InnerKids program	2 sessions of 30 min per week (8 weeks)	Executive functions (inhibition, shifting, emotional control, initiation, working memory, planning/organization, organization of materials, and monitoring)	The mindfulness group exhibited improvements in executive function (overall global executive control, behavioral regulation, and metacognition), when compared with the control group.
Gould et al. (2012)	Yoga-inspired mindfulness program	4 sessions of 45 min per week (12 weeks)	Depressive symptoms, positive and negative emotions, and stress responses	The mindfulness group showed a reduction in “impulsive action stress responses” (in youth who had low baseline depressive symptoms) and reduction in “Involuntary Engagement stress responses” (in youth who had low or medium levels of baseline depressive symptoms), compared with the control group.
Janz et al. (2019)	CalmSpace	Range of mindfulness activities that complement the school routine and curriculum.	Executive functioning	Compared to children in a waitlist control condition, children who participated in the mindfulness program showed improvements in measures of inhibitory control and cognitive flexibility. There were also significant gains in measures of behavior, most notably in attentional control processes.
Parker et al. (2014)	Master Mind program	1 session of 15 min each per week (4 weeks)	Executive functioning (inhibitory control, cognitive flexibility, and working memory), behavior, emotion regulation, and intentions to use substances	The mindful group significantly increased executive functioning skills (girls and boys), self-control abilities (only boys), and significantly reduced aggressive behavior and social problems (boys and girls), and anxiety (only girls), when compared with the control group.
Ricarte et al. (2015)	Mindfulness-based intervention (MBI)	30 sessions of 15 min each (6 weeks)	Mood state, attention, and concentration	The mindfulness group improved mood state, concentration, and immediate auditory-verbal memory, compared with the control group
Rodríguez-Ledo et al. (2018)	Emotional Competency Development SEA program	18 sessions of 55 min (9 months)	Emotional intelligence and mindfulness	The mindfulness group showed a significant effect in the ability to mindfully attend the interior and in the capacity of Kinesthetic attention, compared with the control group.
Schonert-Reichl and Lawlor (2010)	Mindfulness Education (ME) program	10 sessions of 40-50 min each per week + mindfulness attention exercises (3 times a day, at least 3 min each session) (10 weeks)	Optimism, school and general self-concept, positive and negative emotions, classroom social and emotional competence (i.e., aggressive behaviors, oppositional behavior/dysregulation, attention and concentration, social-emotional competence)	The mindful group showed significant increases in social and emotional competence (attention/concentration and social-emotional competence) as well as in positive emotions (i.e., optimism) when compared with the control group. Also, the mindful group demonstrated improvements in general self-concept (for preadolescents, but no for early adolescents), compared with the control group.
Schonert-Reichl et al. (2015)	MindUP program	1 session of 40 – 50 min per week (12 sessions)	Executive functions, stress physiology (through salivary cortisol), wellbeing, empathy, optimism, perspective-taking, emotional control, school self-concept, depressive symptoms, social responsibility, mindfulness, social responsibility, and pro-sociality	Compared with the control group, the mindfulness group showed (a) more improvements in executive functions and stress physiology; (b) higher empathy, perspective-taking, emotional control, optimism, school self-concept, and mindfulness; (c) greater decreases in symptoms of depression and peer aggression; (d) higher rates of prosocial behavior; and (e) increased peer acceptance.
Tarrasch (2018)	Mindfulness-based stress reduction (MBSR)	1 session of approximately 45 min per week (10 weeks)	Sustained and selective attention	A significant improvement in attentional tasks was obtained in the mindful group.
Tarrasch et al. (2017)	Call to Care-Israel	1 session of 45 min per week (24 weeks)	Visual perception, motor accuracy, anxiety, and mindfulness	The mindfulness group showed improvements in motor accuracy, visual perception, and mindfulness and reduction of anxiety, compared with the control group.
Thomas and Atkinson (2016)	Paws.b	6 sessions of 60 min each per week (6 weeks)	Attentional functioning	The mindfulness group had a significant positive impact on children's attentional functioning when compared with the control group.
van de Weijer-Bergsma et al. (2014)	MindfulKids	Twelve 30-min sessions (6 weeks)	Stress and stress-related mental health and behavioral problems	Prevention effects on stress and wellbeing were found directly after training. Effects on mental health problems also became apparent at follow-up.
Viafora et al. (2015)	Mindfulness activities	1 session of 45 min each per week (8 weeks)	Emotional wellbeing, and positive behaviors: self-compassion, mindfulness, psychological acceptance, and psychological inflexibility	The mindful group 1 showed significant improvements in acceptance and mindful awareness, and the mindful group 2 showed higher emotional wellbeing, more facility at dealing with difficult feelings (such as anger, stress), and learned to be more patient.Both mindful groups expressed benefits in various domains, such as concentration, stress, relaxation, patience, happiness, and in the ability to deal with difficult feelings, compared with the control group.
Vickery and Dorjee (2016)	Paws.b	12 sessions of 30 min (8 weeks)	Emotional wellbeing	The mindfulness group showed a significant increase in meta-cognition and significantly reduced negative affect when compared to the control group.
Waldemar et al. (2016)	Mindfulness and social-emotional learnings program (M-SEL)	From 8 to 12 sessions of 60 min (5 months)	Mental health problems (emotional, conduct, hyperactivity, relationship, and prosocial), quality of life, and symptoms of attention deficit hyperactivity disorder	The mindfulness group showed significant improvements in four mental health problems (i.e., emotional problems, conduct problems, interpersonal relationships, and prosocial behavior) as well as in the quality of life, compared with the control group.
White (2012)	The mindful awareness for girls through yoga program	1 session of 60 min per week + 10 min of yoga daily homework (8 weeks)	Perceived stress, coping abilities, self-esteem, and self-regulation	The mindfulness group was more likely to report a higher appraisal of stress and greater frequency of coping, compared with the control group. Self-esteem and self-regulation increased in both groups.
Wimmer et al. (2016)	Mindfulness training-based on MBSR method	2 sessions of 60 and 90 min, respectively, per week (18 weeks)	Sustained attention, cognitive flexibility, cognitive inhibition, and data-driven information processing	The mindfulness group showed improvements in cognitive inhibition and data-driven information processing when compared with both control groups (active and passive). Also, the sustained attention performance of the experimental group was better than the passive control group.
Yook et al. (2017)	New sport and mindfulness yoga (physical activity intervention)	1 new sport session of 40 min + 1 mindfulness yoga session of 40 min per week (8 weeks)	Self-esteem, resilience, and happiness	The mindfulness group exhibited significant improvements in self-esteem and resilience, and significant change in psychological happiness, compared with the control group.

As can be seen in Table 3, the included studies examined several mindfulness interventions that, in most cases, were linked to previously existing mindfulness programs, such as mindfulness-based cognitive therapy (MBCT) for depression or mindfulness-based stress reduction (MBSR). Importantly, only nine programs were assessed with RCTs, namely, the MBCT adapted to the Kamalayan curriculum, the MBSR—adapted program, the Calmer Choice, the Meditation condition formulated according to Roth's Integrative Contemplative Pedagogy, the Kripalu Yoga in the Schools, the Innerkids Program, the Master Mind, the MindUP program, and the Paws.b.

#### Effects of Interventions

Overall, as shown in Table 4, among the included studies, 16 assessed cognitive skills, 21 evaluated socio-emotional abilities, and 3 explored academic-related skills. Within these studies, 100% found significant effects for cognitive skills, 90% showed a significant impact on socio-emotional abilities, and 33% suggested a significant improvement in academic skills.

**Table 4:** Mindfulness meditation techniques and significant cognitive, social-emotional, and academic outcomes of the included studies.

		**Significant outcomes**		
**Study**	Meditation techniques^*^	**Cognitive skills**	**Social-emotional skills**	**Academic skills**
Alampay et al. (2019)	(1) Body-centered meditation (2) Mindful observation	–	**x**	–
Bakosh et al. (2016)	(1) Body-centered meditation (2) Mindful observation (4) Mantra meditation (5) Visual concentration (6) Affect-centered meditation	–	✓	✓
Bakosh et al. (2018)	(1) Body-centered meditation (2) Mindful observation (6) Affect-centered meditation	–	–	**x**
Bauer et al. (2020)	(1) Body-centered meditation (2) Mindful observation (4) Mantra meditation	✓	–	–
Britton et al. (2014)	(1) Body-centered meditation (2) Mindful observation	–	**x**	–
Crescentini et al. (2016)	(1) Body-centered meditation (2) Mindful observation (6) Affect-centered meditation (7) Meditation with movement	✓	✓	–
Bergen-Cico et al. (2015)	(1) Body-centered meditation (2) Mindful observation (7) Meditation with movement	✓	✓	–
Butzer et al. (2017)	(1) Body-centered meditation (2) Mindful observation (6) Affect-centered meditation (7) Meditation with movement	✓	✓	–
de Carvalho et al. (2017)	(1) Body-centered meditation (2) Mindful observation (4) Mantra meditation (5) Visual Concentration (6) Affect-centered meditation (7) Meditation with movement	–	✓	–
Devcich et al. (2017)	(1) Body-centered meditation (2) Mindful observation (4) Mantra meditation (5) Visual concentration (6) Affect-centered meditation (7) Meditation with movement	–	✓	–
Enoch and Dixon (2017)	(1) Body-centered meditation (2) Mindful observation (4) Mantra meditation (5) Visual Concentration	✓	–	–
Flook et al. (2010)	(1) Body-centered meditation (2) Mindful observation (4) Mantra meditation (5) Visual concentration (6) Affect-centered meditation (7) Meditation with movement	✓	–	–
Gould et al. (2012)	(1) Body-centered meditation (2) Mindful observation (6) Affect-centered meditation (7) Meditation with movement	✓	✓	–
Janz et al. (2019)	(1) Body-centered meditation (2) Mindful observation (4) Mantra meditation (5) Visual concentration (7) Meditation with movement	✓	–	–
Parker et al. (2014)	(1) Body-centered meditation (2) Mindful observation (6) Affect-centered meditation (7) Meditation with movement	✓	✓	–
Ricarte et al. (2015)	(1) Body-centered meditation (2) Mindful observation (4) Mantra meditation (5) Visual Concentration	✓	✓	–
Rodríguez-Ledo et al. (2018)	(1) Body-centered meditation (2) Mindful observation	–	✓	–
Schonert-Reichl and Lawlor (2010)	(1) Body-centered meditation (2) Mindful observation (4) Mantra meditation (5) Visual Concentration (6) Affect-centered meditation	–	✓	–
Schonert-Reichl et al. (2015)	(1) Body-centered meditation (2) Mindful observation (4) Mantra meditation (5) Visual concentration (6) Affect-centered meditation	✓	✓	**x**
Tarrasch (2018)	(1) Body-centered meditation (2) Mindful observation (4) Mantra meditation (5) Visual concentration (7) Meditation with movement	✓	–	–
Tarrasch et al. (2017)	(1) Body-centered meditation (2) Mindful observation (4) Mantra meditation (5) Visual concentration (6) Affect-centered meditation (7) Meditation with movement	✓	✓	–
Thomas and Atkinson (2016)	(1) Body-centered meditation (2) Mindful observation	✓	–	–
van de Weijer-Bergsma et al. (2014)	(1) Body-centered meditation (2) Mindful observation (4) Mantra meditation (6) Affect-centered meditation	–	✓	–
Viafora et al. (2015)	(1) Body-centered meditation (2) Mindful observation (4) Mantra meditation (5) Visual concentration (6) Affect-centered meditation (7) Meditation with movement	–	✓	–
Vickery and Dorjee (2016)	(1) Body-centered meditation (2) Mindful observation (6) Affect-centered meditation	✓	✓	–
Waldemar et al. (2016)	(1) Body-centered meditation (2) Mindful observation (4) Mantra meditation (5) Visual concentration (6) Affect-centered meditation	–	✓	–
White (2012)	(1) Body-centered meditation (2) Mindful observation (4) Mantra meditation (5) Visual concentration (7) Meditation with movement	–	✓	–
Wimmer et al. (2016)	(1) Body-centered meditation (2) Mindful observation (7) Meditation with movement	✓	–	–
Yook et al. (2017)	(1) Body-centered meditation (2) Mindful observation (7) Meditation with movement	–	✓	–

* *Meditation techniques classified according to Matko and Sedlmeier (2019): (1) body-centered meditation, (2) mindful observation, (3) contemplation, (4) mantra meditation, (5) visual concentration, (6) affect-centered meditation, and (7) meditation with movement; ✓: significant difference found; x: significant difference not found*.

The RCTs showed effects for measures assessing cognitive (*viz*. executive functions, attention, and self-control) and social-emotional (*viz*. stress physiology, empathy, perspective taking, emotional control, optimism, school self-concept, symptoms of depression, anxiety, peer aggression, prosocial behavior, and peer acceptance) skills (Tables 3, 4).

The QEDs also found positive effects for cognitive skills (i.e., overall executive functions, attention, concentration, inhibitory control, cognitive flexibility, and immediate auditory-verbal memory), social-emotional abilities (*viz*. stress, wellbeing, mindfulness, self-esteem, resilience, psychological happiness, empathy, perspective-taking, emotional control, optimism, symptoms of depression, internalizing problems, peer aggression, prosocial behavior, increased peer acceptance, reduced anxiety, self-control, self-regulation, improvements in mental health problems, quality of life, self-compassion, acceptance, relaxation, happiness, aggressive behaviors, and social competence), and academic skills (*viz*. school self-concept, science and reading grades, and classroom behavior; Table 3).

#### Exploring the Effects of Meditation Techniques on Cognitive, Socio-Emotional, and Academic Skills

Overall, all interventions incorporated “body-centered meditations” and “mindful observations.” Almost half of the interventions included “affect-centered meditations” (55%), “mantra meditations” (55%), “meditations with movement” (52%), and “visual concentration” (48%) (Table 3). Patterns of relationships between meditation techniques and cognitive, socio-emotional, and academic outcomes were identified and are presented below (detailed description presented in Table 4).

#### Cognitive Performance

As detailed in Table 4, results showed that interventions improving cognitive outcomes frequently used “body-centered meditations” and “mindful observations,” while less frequent techniques to improve cognitive abilities were “affect-centered meditations,” “meditations with movement,” “visual concentrations,” and “mantra meditation.” The six RCTs that found improvements in cognitive skills used “body-centered meditations” (*n* = 6), “mindful observations” (*n* = 6), “affect-centered meditations” (*n* = 4), “meditations with movement” (*n* = 3), “mantra meditations” (*n* = 3), and “visual concentration” (*n* = 2). The 10 QEDS that showed benefits in cognitive outcomes employed “body-centered meditations” (*n* = 10), “mindful observations” (*n* = 10), “meditations with movement” (*n* = 7), “mantra meditations” (*n* = 5), “visual concentration” (*n* = 5), and “affect-centered meditations” (*n* = 4) (Table 4).

#### Socio-emotional Abilities

Findings suggested that, although all the interventions improving socio-emotional outcomes included “body-centered meditations” and “mindful observations,” “affect-centered meditations” were also frequently applied. Less frequent techniques used to improve socio-emotional outcomes were “meditations with movement,” “visual concentrations,” and “mantra meditation.” Three RCTs found improvements in socio-emotional abilities and applied “body-centered meditations” (*n* = 3), “mindful observations” (*n* = 3), “affected-centered meditations” (*n* = 3), “meditations with movement” (*n* = 2), “mantra meditation” (*n* = 1), and “visual concentration” (*n* = 1). Sixteen QEDs also showed benefits in socio-emotional skills and used “body-centered meditations” (*n* = 16), “mindful observations” (*n* = 16), “mantra meditations” (*n* = 10), “visual concentration” (*n* = 10), “affect-centered meditations” (*n* = 10), and “meditations with movements” (*n* = 10) (Table 4).

#### Academic Skills

Just one QED found improvements in academic skills. This study used “body-centered meditations,” “mindful observations,” “mantra meditations,” “visual concentration,” and “affect-centered meditations” (Table 4).

## Discussion

To the best of our knowledge, this is the first review of the efficacy of specific meditation techniques used by mindfulness-based programs on the cognitive, social-emotional, and academic skills of children. A total of 29 studies (nine RCTs and 20 QED studies) met the selection criteria. Overall, results provided support for the use of mindfulness interventions to improve cognitive and social-emotional outcomes but found no support for the use of these interventions to enhance academic skills. Regarding meditation mindfulness techniques, all the interventions used “body-centered meditations” and “mindful observations.” In addition, “affect-centered meditations” were also frequently applied to improve socio-emotional outcomes. Less frequent techniques were “meditations with movements,” “mantra meditation,” and “visual concentration.” Thus, the effective techniques used in mindfulness-based programs differ in terms of activation and amount of body orientation, dimensions that highlight the role of embodied cognition in meditation. The most frequent techniques for improving cognitive and socio-emotional outcomes were active practices with a lower amount of body orientation. For socio-emotional outcomes, effective practices also included a higher abstract and conceptual focus and a neutral amount of body orientation. However, given the high risk of bias across the included studies in several domains, caution is needed in interpreting the results.

The findings of the present review support the favorable impacts of mindfulness interventions on cognitive outcomes, as expected (Flook et al., 2010). The next largest area of impact was related to socio-emotional skills. Indeed, our findings show that many of the outcomes were linked to emotional regulation processes involved in mindfulness training that also correspond to what was expected and highlighted in previous research (Bohlmeijer et al., 2010; Fjorback et al., 2011; Gotink et al., 2015; Guendelman et al., 2017).

Among the included studies, the specific techniques used to improve cognitive and socio-emotional outcomes were very similar, that is, all the interventions used “body-centered meditations” and “mindful observations.” There are several ways in which this pattern of results can be explained. In fact, “body-centered meditations” and “mindful observations” may be more effective than other techniques in improving cognitive and socio-emotional outcomes given that they provide more explicit instructions, possibly making it easier for children to use upon times of strong emotions. For instance, research showed that the redirection of attention to the body can improve attention, regulate stress, and enable a deeper understanding of our emotional-motivational state (Bornemann et al., 2015; Fissler et al., 2016). The body scan (i.e., focusing attention sequentially on various parts of the body) is another meditation frequently included in these clusters of techniques. This practice was associated with the components of observing and non-reacting, promoting wellbeing, and decreasing anxiety (Carmody and Baer, 2009).

In addition, “affect-centered meditations” appear to be a good strategy for improving socio-emotional skills that includes positive feelings and kindness, cultivates self-care, or provides meaningful experiences of connection with others. Previous research has shown that after this kind of practice, adult participants revealed a significant change in brain regions previously linked with empathy, compassion, and emotion regulation (Klimecki et al., 2012). Also, it seems that practices focused on affect and perspective taking produced significant decreases in the release of cortisol (i.e., a stress hormone), suggesting that this component may be associated with a significant reduction in physiological stress (Engert et al., 2017).

Regarding academic skills, although previous research suggested positive effects (McCloskey, 2015; Lin and Mai, 2018), just three QED studies included in this review aimed at investigating this domain, which makes it difficult to draw conclusions related to mindfulness techniques. However, the effects found for cognitive and socio-emotional outcomes might be related to the measures that are typically used to assess these particular skills (i.e., self-reports versus the administrative measures used to evaluate academic achievements; Maynard et al., 2017).

Our findings highlight the need for examining the unique contribution of intervention components in mindfulness-based interventions, as suggested by previous research. For instance, Carmody and Baer (2008) reported that practicing mindfulness movement (yoga), but not sitting meditation and body scan, was associated with higher levels of nonjudgment of inner experience. Thus, indeed, different mindfulness practices may target different aspects of psychological health.

Previous research suggests that one main factor associated with the variable results across studies is the amount of mindfulness practice introduced (Zenner et al., 2014). Our findings also highlighted the idea that the optimal meditation duration, intensity, and dosage for children are still unknown. In the included studies, children meditated, approximately, from 4 to 90 min per session, from 2 weeks to 9 months. Thus, the dosage and frequency of mindfulness meditation varied significantly between studies, and the total time meditating may be related to cognitive or emotional changes. Some studies appear to have reduced the amount of meditation time when compared with mindfulness interventions for adults, which commonly involve as much as 45 min of practice per day (Teasdale et al., 2000; Segal et al., 2002). Also, some mindfulness interventions with adolescents found effects with 20 or more minutes of practice per day (Saltzman and Goldin, 2008; Biegel et al., 2009). Still, other studies have reported significant effects of mindfulness intervention in children and adolescents with just 5 min of daily meditation (Saltzman and Goldin, 2008; Zylowska et al., 2008; Britton et al., 2010). Thus, further research should examine the impact of increased mindfulness meditation time, and this issue deserves more systematic investigation (Greenberg and Harris, 2012).

Despite these results showing that specific mindfulness training techniques can have different benefits for children, limitations of our findings should be reported: (i) although we reported study characteristics that are indicators of study quality, the risk of bias assessment was conducted only for randomized studies; (ii) the heterogeneity of the studies is considerable, and due to the vast array of practices, this review only examined clusters of techniques; and (iii) the frequent lack of blinded raters, randomization, active comparison groups, and small samples of the included studies mitigate the impact of our findings. Future studies must address these issues to support empirical evidence about the effect of mindfulness techniques on the development of children. A well-designed intervention should have the following key features: (a) randomization of participants into the experimental and control groups; (b) control for participants and expectations of an informant through blinding and assessment of expectations before the beginning of the intervention to control for possible placebo effects; and (c) comparison of the performance of the experimental group to both active and passive control conditions (e.g., Kendall, 2003).

Furthermore, a content analysis across the included studies would be useful to conduct an in-depth review of specific mindfulness techniques. Through this particular approach, methodological rigor is increased as qualitative data are categorized deductively or inductively (Forman and Damschroder, 2007).

Another area to further develop is not only to examine which mindfulness techniques are effective but also to understand which components are necessary (e.g., does movement enhance mindfulness practice?) and what works for whom. Indeed, studies have found that baseline characteristics predict intervention outcomes (Cordon et al., 2009) and that mindfulness-based interventions may be ineffective (e.g., Jazaieri et al., 2012) or contraindicated for specific conditions (Ma and Teasdale, 2004; Arch and Ayers, 2013). Regarding children and adolescents, the effects of mindfulness-based training programs have been associated with preexisting characteristics, such as levels of executive function, age, and family environment (e.g., Barnes et al., 2010; Flook et al., 2010; Schonert-Reichl and Lawlor, 2010). Therefore, researchers need to be aware of the possibility of both positive and adverse effects that certain practices could have on children with different characteristics (Greenberg and Harris, 2012).

Finally, since mindfulness-based programs consist of a variety of techniques, there may be elements other than the mindfulness component that are effective. So, an important distinction to be further explored is the unique effect of specific mindfulness exercises apart from other meditative practices.

Overall, this study represents a preliminary attempt to isolate the effects of different meditation techniques on the positive outcomes associated with cognitive, socio-emotional, and academic skills. Although in the past many studies on mindfulness training can be criticized for their lack of scientific rigor (Toneatto and Nguyen, 2007; Chiesa and Serretti, 2011), more recent studies provide strong evidence for the utility of such interventions, and it is hoped that these data encourage further studies on the unique effects of different mindfulness techniques.

## Data Availability Statement

The raw data supporting the conclusions of this article will be made available by the authors, without undue reservation, to any qualified researcher.

## Author Contributions

MF, SM, PA, SC, and TL contributed to the conception and design of the manuscript. MF, SM, AV, AC, and LR were responsible for the acquisition of data. MF, SM, and PA contributed to the interpretation of data. MF wrote the first draft of the manuscript. All authors contributed to manuscript revision and approved the submitted version.

## Funding

This research was supported by the Portuguese Foundation for Science and Technology (FCT; Grants 2020.01866.CEECIND, UID/PSI/00050/2013) and was conducted within the M2S Project funded through the Operational Programme for Competitiveness and Internationalization, supported by FEDER and national funds allocated to FCT (NORTE-01-0145-FEDER-028404).

## Conflict of Interest

The authors declare that the research was conducted in the absence of any commercial or financial relationships that could be construed as a potential conflict of interest.

## Publisher's Note

All claims expressed in this article are solely those of the authors and do not necessarily represent those of their affiliated organizations, or those of the publisher, the editors and the reviewers. Any product that may be evaluated in this article, or claim that may be made by its manufacturer, is not guaranteed or endorsed by the publisher.

## References

[B1] Alampay L. P. Galvez Tan L. J. T. Tuliao A. P. Baranek P. Ofreneo M. A. Lopez G. D. . (2019). A pilot randomized controlled trial of a mindfulness program for Filipino children. Mindfulness 11, 303–316. 10.1007/s12671-019-01124-8

[B2] Arch J. J. Ayers C. R. (2013). Which treatment worked better for whom? moderators of group cognitive behavioral therapy versus adapted mindfulness based stress reduction for anxiety disorders. Behav. Res. Ther. 51, 434–442. 10.1016/j.brat.2013.04.004 23747582

[B3] Baer R. A. (2003). Mindfulness training as a clinical intervention: a conceptual and empirical review. Clin. Psychol. Sci. Pract. 10, 125–143. 10.1093/clipsy.bpg015

[B4] Bakosh L. S. Snow R. M. Tobias J. M. Houlihan J. L. Barbosa-Leiker C. (2016). Maximizing mindful learning: mindful awareness intervention improves elementary school students' quarterly grades. Mindfulness 7, 59–67. 10.1007/s12671-015-0387-6

[B5] Bakosh L. S. Tobias Mortlock J. M. Querstret D. Morison L. (2018). Audio-guided mindfulness training in schools and its effect on academic attainment: contributing to theory and practice. Learn. Instr. 58, 34–41. 10.1016/j.learninstruc.2018.04.012

[B6] Barnes V. A. Gregoski M. J. Tingen M. S. Treiber F. A. (2010). Influences of family environment and meditation efficacy on hemodynamic function among African American adolescents. J. Complement. Integr. Med. 7:1326. 10.2202/1553-3840.1326 22328869 PMC3276075

[B7] Bauer C. C. C. Rozenkrantz L. Caballero C. Nieto-Castanon A. Scherer E. West M. R. . (2020). Mindfulness training preserves sustained attention and resting state anticorrelation between default-mode network and dorsolateral prefrontal cortex: a randomized controlled trial. Hum. Brain Mapp. 41, 5356–5369. 10.1002/hbm.25197 32969562 PMC7670646

[B8] Bergen-Cico D. Razza R. Timmins A. (2015). Fostering self-regulation through curriculum infusion of mindful yoga: a pilot study of efficacy and feasibility. J. Child Fam. Stud. 24, 3448–3461. 10.1007/s10826-015-0146-2

[B9] Biegel G. M. Brown K. W. Shapiro S. L. Schubert C. M. (2009). Mindfulness-based stress reduction for the treatment of adolescent psychiatric outpatients: A randomized clinical trial. J. Consult. Clin. Psychol. 77, 855–866. 10.1037/a0016241 19803566

[B10] Bishop S. R. Lau M. Shapiro S. Carlson L. Anderson N. D. Carmody J. . (2004). Mindfulness: A proposed operational definition. Clin. Psychol. Sci. Pract. 11, 230–241. 10.1093/clipsy.bph077

[B11] Bohlmeijer E. Prenger R. Taal E. Cuijpers P. (2010). The effects of mindfulness-based stress reduction therapy on mental health of adults with a chronic medical disease: a meta-analysis. J. Psychosom. Res. 68, 539–544. 10.1016/j.jpsychores.2009.10.005 20488270

[B12] Bornemann B. Herbert B. M. Mehling W. E. Singer T. (2015). Differential changes in self-reported aspects of interoceptive awareness through 3 months of contemplative training. Front. Psychol. 5:1504. 10.3389/fpsyg.2014.01504 25610410 PMC4284997

[B13] Bowen S. Witkiewitz K. Dillworth T. M. Chawla N. Simpson T. L. Ostafin B. D. . (2006). Mindfulness meditation and substance use in an incarcerated population. Psychol. Addict. Behav. 20, 343–347. 10.1037/0893-164X.20.3.343 16938074

[B14] Britton W. B. Bootzin R. R. Cousins J. C. Hasler B. P. Peck T. Shapiro S. L. (2010). The contribution of mindfulness practice to a multicomponent behavioral sleep intervention following substance abuse treatment in adolescents: a treatment-development study. Subst. Abus. 31, 86–97. 10.1080/08897071003641297 20408060

[B15] Britton W. B. Lepp N. E. Niles H. F. Rocha T. Fisher N. E. Gold J. S. (2014). A randomized controlled pilot trial of classroom-based mindfulness meditation compared to an active control condition in sixth-grade children. J. Sch. Psychol. 52, 263–278. 10.1016/j.jsp.2014.03.002 24930819 PMC4060047

[B16] Butzer B. LoRusso A. Shin S. H. Khalsa S. B. S. (2017). Evaluation of yoga for preventing adolescent substance use risk factors in a middle school setting: a preliminary group-randomized controlled trial. J. Youth Adolesc. 46, 603–632. 10.1007/s10964-016-0513-3 27246653 PMC5133199

[B17] Carmody J. Baer R. A. (2008). Relationships between mindfulness practice and levels of mindfulness, medical and psychological symptoms and well-being in a mindfulness-based stress reduction program. J. Behav. Med. 31, 23–33. 10.1007/s10865-007-9130-7 17899351

[B18] Carmody J. Baer R. A. (2009). How long does a mindfulness-based stress reduction program need to be? a review of class contact hours and effect sizes for psychological distress. J. Clin. Psychol. 65, 627–638. 10.1002/jclp.20555 19309694

[B19] Chiesa A. (2009). Zen meditation: an integration of current evidence. J. Altern. Complement. Med. 15, 585–592. 10.1089/acm.2008.0416 19422285

[B20] Chiesa A. Serretti A. (2011). Mindfulness based cognitive therapy for psychiatric disorders: a systematic review and meta-analysis. Psychiatry Res. 187, 441–453. 10.1016/j.psychres.2010.08.011 20846726

[B21] Cordon S. L. Brown K. W. Gibson P. R. (2009). The role of mindfulness-based stress reduction on perceived stress: preliminary evidence for the moderating role of attachment style. J. Cogn. Psychother. 23, 258–269. 10.1891/0889-8391.23.3.258

[B22] Crescentini C. Capurso V. Furlan S. Fabbro F. (2016). Mindfulness-oriented meditation for primary school children: Effects on attention and psychological well-being. Front. Psychol. 7:805. 10.3389/fpsyg.2016.00805 27375510 PMC4894866

[B23] Davidson R. J. Kaszniak A. W. (2015). Conceptual and methodological issues in research on mindfulness and meditation. Am. Psychol. 70, 581–592. 10.1037/a0039512 26436310 PMC4627495

[B24] de Carvalho J. S. Pinto A. M. Marôco J. (2017). Results of a mindfulness-based social-emotional learning program on portuguese elementary students and teachers: a quasi-experimental study. Mindfulness 8, 337–350. 10.1007/s12671-016-0603-z

[B25] Devcich D. A. Rix G. Bernay R. Graham E. (2017). Effectiveness of a mindfulness-based program on school children's self-reported well-being: a pilot study comparing effects with an emotional literacy program. J. Appl. Sch. Psychol. 33, 309–330. 10.1080/15377903.2017.1316333

[B26] Docksai R. (2013). A mindful approach to learning. Futurist 47, 8–10.

[B27] Engert V. Kok B. E. Papassotiriou I. Chrousos G. P. Singer T. (2017). Specific reduction in cortisol stress reactivity after social but not attention-based mental training. Sci. Adv. 3:e1700495. 10.1126/sciadv.1700495 28983508 PMC5627978

[B28] Enoch M. R. Dixon M. R. (2017). The use of a child-based acceptance and commitment therapy curriculum to increase attention. Child Fam. Behav. Ther. 39, 200–224. 10.1080/07317107.2017.1338454

[B29] Felver J. C. Tipsord J. M. Morris M. J. Racer K. H. Dishion T. J. (2017). The effects of mindfulness-based intervention on children's attention regulation. J. Atten. Disord. 21, 872–881. 10.1177/1087054714548032 25172884

[B30] Fissler M. Winnebeck E. Schroeter T. Gummersbach M. Huntenburg J. M. Gaertner M. . (2016). An investigation of the effects of brief mindfulness training on self-reported interoceptive awareness, the ability to decenter, and their role in the reduction of depressive symptoms. Mindfulness 7, 1170–1181. 10.1007/s12671-016-0559-z

[B31] Fjorback L. O. Arendt M. Ørnbøl E. Fink P. Walach H. (2011). Mindfulness-based stress reduction and mindfulness-based cognitive therapy—a systematic review of randomized controlled trials. Acta Psychiatr. Scand. 124, 102–119. 10.1111/j.1600-0447.2011.01704.x 21534932

[B32] Flook L. Smalley S. L. Kitil M. J. Galla B. M. Kaiser-Greenland S. Locke J. . (2010). Effects of mindful awareness practices on executive functions in elementary school children. J. Appl. Sch. Psychol. 26, 70–95. 10.1080/15377900903379125

[B33] Forman J. Damschroder L. (2007). Qualitative content analysis, in Empirical methods for bioethics: A prime, eds. L. Jacoby and L. A. Siminoff (Emerald Group Publishing Limited), 39–62.

[B34] Goleman D. (1988). The meditative mind: The varieties of meditative experience. Putnam.

[B35] Goleman D. Davidson R. J. (2018). Traços alterados. Círculo Leitores.

[B36] Gotink R. A. Chu P. Busschbach J. J. V. Benson H. Fricchione G. L. Hunink M. G. M. (2015). Standardised mindfulness-based interventions in healthcare: an overview of systematic reviews and meta-analyses of RCTs. PLoS ONE 10:e0124344. 10.1371/journal.pone.0124344 25881019 PMC4400080

[B37] Gould L. F. Dariotis J. K. Mendelson T. Greenberg M. T. (2012). A school-based mindfulness intervention for urban youth: exploring moderators of intervention effects. J. Community Psychol. 40, 968–982. 10.1002/jcop.21505

[B38] Greenberg M. T. Harris A. R. (2012). Nurturing mindfulness in children and youth: current state of research. Child Dev. Perspect. 6, 161–166. 10.1111/j.1750-8606.2011.00215.x

[B39] Grossman P. Niemann L. Schmidt S. Walach H. (2004). Mindfulness-based stress reduction and health benefits: a meta-analysis. J. Psychosom. Res. 57, 35–43. 10.1016/S0022-3999(03)00573-7 15256293

[B40] Guendelman S. Medeiros S. Rampes H. (2017). Mindfulness and emotion regulation: Insights from neurobiological, psychological, and clinical studies. Front. Psychol. 8:220. 10.3389/fpsyg.2017.00220 28321194 PMC5337506

[B41] Hildebrandt L. K. McCall C. Singer T. (2017). Differential effects of attention-, compassion-, and socio-cognitively based mental practices on self-reports of mindfulness and compassion. Mindfulness. 8, 1488–1512. 10.1007/s12671-017-0716-z 29201246 PMC5693975

[B42] Huppert F. A. Johnson D. M. (2010). A controlled trial of mindfulness training in schools: The importance of practice for an impact on well-being. J. Posit. Psychol. 5, 264–274. 10.1080/17439761003794148

[B43] Janz P. Dawe S. Wyllie M. (2019). Mindfulness-based program embedded within the existing curriculum improves executive functioning and behavior in young children: a waitlist controlled trial. Front. Psychol. 10:2052. 10.3389/fpsyg.2019.02052 31551884 PMC6746974

[B44] Jazaieri H. Goldin P. R. Werner K. Ziv M. Gross J. J. (2012). A randomized trial of MBSR versus aerobic exercise for social anxiety disorder. J. Clin. Psychol. 68, 715–731. 10.1002/jclp.21863 22623316 PMC4136448

[B45] Kabat-Zinn J. (2003). Mindfulness-based interventions in context: past, present, and future. Clin. Psychol. Sci. Pract. 10, 144–156. 10.1093/clipsy.bpg016

[B46] Kendall J. M. (2003). Designing a research project: randomised controlled trials and their principles. Emerg. Med. J. 20, 164–168. 10.1136/emj.20.2.164 12642531 PMC1726034

[B47] Keng S.-L. Smoski M. J. Robins C. J. (2011). Effects of mindfulness on psychological health: a review of empirical studies. Clin. Psychol. Rev. 31, 1041–1056. 10.1016/j.cpr.2011.04.006 21802619 PMC3679190

[B48] Klimecki O. M. Leiberg S. Lamm C. Singer T. (2012). Functional neural plasticity and associated changes in positive affect after compassion training. Cereb. Cortex 23, 1552–1561. 10.1093/cercor/bhs142 22661409

[B49] Klingbeil D. A. Renshaw T. L. Willenbrink J. B. Copek R. A. Chan K. T. Haddock A. . (2017). Mindfulness-based interventions with youth: a comprehensive meta-analysis of group-design studies. J. Sch. Psychol. 63, 77–103. 10.1016/j.jsp.2017.03.006 28633940

[B50] Lawler J. M. Esposito E. A. Doyle C. M. Gunnar M. R. (2019). A preliminary, randomized-controlled trial of mindfulness and game-based executive function trainings to promote self-regulation in internationally-adopted children. Dev. Psychopathol. 31, 1513–1525. 10.1017/S0954579418001190 30698120

[B51] Ledesma D. Kumano H. (2009). Mindfulness-based stress reduction and cancer: a meta-analysis. Psychooncology. 18, 571–579. 10.1002/pon.1400 19023879

[B52] Leonard N. R. Jha A. P. Casarjian B. Goolsarran M. Garcia C. Cleland C. M. . (2013). Mindfulness training improves attentional task performance in incarcerated youth: A group randomized controlled intervention trial. Front. Psychol. 4:792. 10.3389/fpsyg.2013.00792 24265621 PMC3820955

[B53] Lin J. W. Mai L. J. (2018). Impact of mindfulness meditation intervention on academic performance. Innov. Educ. Teach. Int. 55, 366–375. 10.1080/14703297.2016.1231617

[B54] Lutz A. Jha A. P. Dunne J. D. Saron C. D. (2015). Investigating the phenomenological matrix of mindfulness-related practices from a neurocognitive perspective. Am. Psychol. 70, 632–658. 10.1037/a0039585 26436313 PMC4608430

[B55] Ma S. H. Teasdale J. D. (2004). Mindfulness-based cognitive therapy for depression: replication and exploration of differential relapse prevention effects. J. Consult. Clin. Psychol. 72, 31–40. 10.1037/0022-006X.72.1.31 14756612

[B56] Mackenzie M. J. Carlson L. E. Speca M. (2005). Mindfulness-based stress reduction (MBSR) in oncology: rationale and review. Evidence-Based Integr. Med. 2, 139–145. 10.2165/01197065-200502030-00005

[B57] Matchim Y. Armer J. M. (2007). Measuring the psychological impact of mindfulness meditation on health among patients with cancer: a literature review. Oncol. Nurs. Forum 34, 1059–1066. 10.1188/07.ONF.1059-1066 17878133

[B58] Matko K. Sedlmeier P. (2019). What is meditation? proposing an empirically derived classification system. Front. Psychol. 10:2276. 10.3389/fpsyg.2019.02276 31681085 PMC6803504

[B59] Maynard B. R. Solis M. R. Miller V. L. Brendel K. E. (2017). Mindfulness-based interventions for improving cognition, academic achievement, behavior, and socioemotional functioning of primary and secondary school students. Campbell Syst. Rev. 13, 1–144. 10.1002/CL2.177

[B60] McCloskey L. E. (2015). Mindfulness as an intervention for improving academic success among students with executive functioning disorders. Procedia—Soc. Behav. Sci. 174, 221–226. 10.1016/j.sbspro.2015.01.650

[B61] Mendelson T. Greenberg M. T. Dariotis J. K. Gould L. F. Rhoades B. L. Leaf P. J. (2010). Feasibility and preliminary outcomes of a school-based mindfulness intervention for urban youth. J. Abnorm. Child Psychol. 38, 985–994. 10.1007/s10802-010-9418-x 20440550

[B62] Moher D. Liberati A. Tetzlaff J. Altman D. G. The Prisma Group (2009). Preferred reporting items for systematic reviews and meta-analyses: the PRISMA statement. PLoS Med. 6:e1000097. 10.1371/journal.pmed.1000097 21603045 PMC3090117

[B63] Mrazek M. D. Franklin M. S. Phillips D. T. Baird B. Schooler J. W. (2013). Mindfulness training improves working memory capacity and GRE performance while reducing mind wandering. Psychol. Sci. 24, 776–781. 10.1177/0956797612459659 23538911

[B64] Napoli M. Krech P. R. Holley L. C. (2005). Mindfulness training for elementary school students: The attention academy. J. Appl. Sch. Psychol. 21, 99–125. 10.1300/J370v21n01_05

[B65] Nash J. D. Newberg A. (2013). Toward a unifying taxonomy and definition for meditation. Front. Psychol. 4:806. 10.3389/fpsyg.2013.00806 24312060 PMC3834522

[B66] Ott M. J. Norris R. L. Bauer-Wu S. M. (2006). Mindfulness meditation for oncology patients: A discussion and critical review. Integr. Cancer Ther. 5, 98–108. 10.1177/1534735406288083 16685074

[B67] Ouzzani M. Hammady H. Fedorowicz Z. Elmagarmid A. (2016). Rayyan—a web and mobile app for systematic reviews. Syst. Rev. 5:210. 10.1186/s13643-016-0384-4 27919275 PMC5139140

[B68] Parker A. E. Kupersmidt J. B. Mathis E. T. Scull T. M. Sims C. (2014). The impact of mindfulness education on elementary school students: evaluation of the master mind program. Adv. Sch. Ment. Health Promot. 7, 184–204. 10.1080/1754730X.2014.916497 27057208 PMC4821437

[B69] Praissman S. (2008). Mindfulness-based stress reduction: A literature review and clinician's guide. J. Am. Acad. Nurse Pract. 20, 212–216. 10.1111/j.1745-7599.2008.00306.x 18387018

[B70] Quan L. Yanan S. Bin L. Tingyong F. (2019). Mindfulness training can improve 3-and 4-year-old children's attention and executive function. Acta Psychol. Sin. 51:324. 10.3724/SP.J.1041.2019.00324

[B71] Ricarte J. J. Ros L. Latorre J. M. Beltrán M. T. (2015). Mindfulness-based intervention in a rural primary school: effects on attention, voncentration and mood. Int. J. Cogn. Ther. 8, 258–270. 10.1521/ijct_2015_8_03

[B72] Rodríguez-Ledo C. Orejudo S. Cardoso M. J. Balaguer Á. Zarza-Alzugaray J. (2018). Emotional intelligence and mindfulness: relation and enhancement in the classroom with adolescents. Front. Psychol. 9:2162. 10.3389/fpsyg.2018.02162 30473674 PMC6237843

[B73] Saltzman A. Goldin P. (2008). Mindfulness based stress reduction for school-age children, in Acceptance and mindfulness interventions for children and adolescents, eds. L. A. Greco and S. C. Hayes (Oakland, CA: New Harbinger and Context Press), 139–161.

[B74] Schmidt S. (2014). Opening up meditation for science: The ddevelopment of a meditation classification system, in Meditation – Neuroscientific Approaches and Philosophical Implications., eds. S. Schmidt and H. Walach (Springer), 137–152.

[B75] Schonert-Reichl K. A. Lawlor M. S. (2010). The effects of a mindfulness-based education program on pre- and early adolescents' well-being and social and emotional competence. Mindfulness 1, 137–151. 10.1007/s12671-010-0011-8

[B76] Schonert-Reichl K. A. Oberle E. Lawlor M. S. Abbott D. Thomson K. Oberlander T. F. . (2015). Enhancing cognitive and social–emotional development through a simple-to-administer mindfulness-based school program for elementary school children: A randomized controlled trial. Dev. Psychol. 51, 52–66. 10.1037/a0038454 25546595 PMC4323355

[B77] Segal Z. Williams J. Teasdale J. (2002). Mindfulness-based Cognitive Therapy for Depression: A New Approach to Preventing Relapse. New York, NY: Guilford Press.

[B78] Semple R. J. Lee J. Rosa D. Miller L. F. (2010). A randomized trial of mindfulness-based cognitive therapy for children: promoting mindful attention to enhance social-emotional resiliency in children. J. Child Fam. Stud. 19, 218–229. 10.1007/s10826-009-9301-y

[B79] Shapiro S. L. Carlson L. E. (2009). The art and science of mindfulness: Integrating mindfulness into psychology and the helping professions. Am. Psychol. Assoc. 11:885. 10.1037/11885-000

[B80] Singer T. Kok B. E. Bornemann B. Zurborg S. Bolz M. Bochow C. A. (2016). The ReSource Project: Background, design*, samples*, and measurements, in Max Planck Institute for Human Cognitive and Brain Sciences, 11–21.

[B81] Smith J. E. Richardson J. Hoffman C. Pilkington K. (2005). Mindfulness-based stress reduction as supportive therapy in cancer care: systematic review. J. Adv. Nurs. 52, 315–327. 10.1111/j.1365-2648.2005.03592.x 16194185

[B82] Sterne J. A. C. Savovi,ć J. Page M. J. Elbers R. G. Blencowe N. S. Boutron I. . (2019). RoB 2: a revised tool for assessing risk of bias in randomised trials. BMJ 366:14898. 10.1136/bmj.l4898 31462531

[B83] Tarrasch R. (2018). The effects of mindfulness practice on attentional functions among primary school children. J. Child Fam. Stud. 27, 2632–2642. 10.1007/s10826-018-1073-9

[B84] Tarrasch R. Margalit-Shalom L. Berger R. (2017). Enhancing visual perception and motor accuracy among school children through a mindfulness and compassion program. Front. Psychol. 8:281. 10.3389/fpsyg.2017.00281 28286492 PMC5323376

[B85] Teasdale J. D. Segal Z. V. Williams J. M. G. Ridgeway V. A. Soulsby J. M. Lau M. A. (2000). Prevention of relapse/recurrence in major depression by mindfulness-based cognitive therapy. J. Consult. Clin. Psychol. 68, 615–623. 10.1037/0022-006X.68.4.615 10965637

[B86] Teixeira M. E. (2008). Meditation as an intervention for chronic pain. Holist. Nurs. Pract. 22, 225–234. 10.1097/01.HNP.0000326006.65310.a7 18607236

[B87] Thomas G. Atkinson C. (2016). Measuring the effectiveness of a mindfulness-based intervention for children's attentional functioning. Educ. Child Psychol. 33, 51–64. Available online at: https://www.researchgate.net/publication/293826420_Measuring_the_effectiveness_of_a_mindfulness-based_intervention_for_children's_attentional_functioning

[B88] Toneatto T. Nguyen L. (2007). Does mindfulness meditation improve anxiety and mood symptoms? a review of the controlled research. Can. J. Psychiatry 52, 260–266. 10.1177/070674370705200409 17500308

[B89] van de Weijer-Bergsma E. Langenberg G. Brandsma R. Oort F. J. Bögels S. M. (2014). The effectiveness of a school-based mindfulness training as a program to prevent stress in elementary school children. Mindfulness 5, 238–248. 10.1007/s12671-012-0171-9

[B90] Viafora D. P. Mathiesen S. G. Unsworth S. J. (2015). Teaching mindfulness to middle school students and homeless youth in school classrooms. J. Child Fam. Stud. 24, 1179–1191. 10.1007/s10826-014-9926-3

[B91] Vickery C. E. Dorjee D. (2016). Mindfulness training in primary schools decreases negative affect and increases meta-cognition in children. Front. Psychol. 6:2025. 10.3389/fpsyg.2015.02025 26793145 PMC4709470

[B92] Waldemar J. O. C. Rigatti R. Menezes C. B. Guimarães G. Falceto O. Heldt E. (2016). Impact of a combined mindfulness and social–emotional learning program on fifth graders in a Brazilian public school setting. Psychol. Neurosci. 9, 79–90. 10.1037/pne0000044

[B93] White L. S. (2012). Reducing stress in school-age girls through mindful yoga. J. Pediatr. Heal. Care 26, 45–56. 10.1016/j.pedhc.2011.01.002 22153143

[B94] Wimmer L. Bellingrath S. von Stockhausen L. (2016). Cognitive effects of mindfulness training: results of a pilot study based on a theory driven approach. Front. Psychol. 7:1037. 10.3389/fpsyg.2016.01037 27462287 PMC4940413

[B95] Winbush N. Y. Gross C. R. Kreitzer M. J. (2007). The effects of mindfulness-cased stress reduction on sleep disturbance: a systematic review. EXPLORE 3, 585–591. 10.1016/j.explore.2007.08.003 18005910

[B96] Yook Y.-S. Kang S.-J. Park I. (2017). Effects of physical activity intervention combining a new sport and mindfulness yoga on psychological characteristics in adolescents. Int. J. Sport Exerc. Psychol. 15, 109–117. 10.1080/1612197X.2015.1069878

[B97] Zenner C. Herrnleben-Kurz S. Walach H. (2014). Mindfulness-based interventions in schools - A systematic review and meta-analysis. Front. Psychol. 5:603. 10.3389/fpsyg.2014.00603 25071620 PMC4075476

[B98] Zylowska L. Ackerman D. L. Yang M. H. Futrell J. L. Horton N. L. Hale T. S. . (2008). Mindfulness meditation training in adults and adolescents with ADHD. J. Atten. Disord. 11, 737–746. 10.1177/1087054707308502 18025249

